# Identifying the princes base on Altmetrics: An awakening mechanism of sleeping beauties from the perspective of social media

**DOI:** 10.1371/journal.pone.0241772

**Published:** 2020-11-25

**Authors:** Jianhua Hou, Hao Li, Yang Zhang

**Affiliations:** School of Information Management, Sun Yat-sen University, Panyu District, Guangzhou, Guangdong, China; Federation University Australia, AUSTRALIA

## Abstract

In science, sleeping beauties (SBs) denotes a special phenomenon of the diffusion of scientific knowledge based on citation trajectories, the awakening of which is also measured through changes in the citations index. However, the rapid advancement of social media has altered the mode of scientific communication and knowledge diffusion. This study aims to re-identify SBs and its Prince from the perspective of comprehensive indicators, which involves the analysis of Altmetrics indexes and Citation index, and investigate the awakening mechanism of A-SB to supplement the research on the awakening mechanism of SBs. By combining Ab index, we redefined the Prince, which makes A-SB receive high attention after a long Sleeping period and reflects the most prominent academic or social behavior that awakens and sustains the Awakening of A-SB. Then we conducted empirical research on the retrieved *PLOS Biology* collection and examined Prince after identifying the A-SB. The analysis and summary of the characteristics of the identified A-SB and Prince revealed the SBs’ awakening mechanism under the comprehensive trajectory based on Altmetrics from the three dimensions of the influence between the indicators, the overall evolution trajectory of A-SB, and literature bibliometric attributes. In the trajectory of Delayed Recognition stage of A-SB, we define the Dogsleep of SBs, which mirrors that the instability of the Sleeping of SBs will generate a specific negative impact on Prince of A-SB and Awakening intensity. Besides, the literature bibliometric attributes cannot reflect the tendency of users to read academic papers, which again proves that the traditional citation index cannot be neglected in the awakening mechanism of A-SB. Overall, this study demonstrates the addition of the Altmetrics indexes as a useful complement, illustrating the inheritance and connection between the SBs based on the comprehensive trajectory and the SBs based on the citation diffusion trajectory.

## Introduction

Van Raan referred to the phenomenon that scientific literature was not cited for a prolonged time after publication (“Sleeping”) and then suddenly received numerous citations (“Awakened by Prince”) as “Scientific Sleeping Beauty” (SBs) [1]. Then, Li & Ye proposed “All-Elements Scientific Sleeping Beauty” to supplement the concept discussed above [2]. Nevertheless, Prince literature is a necessity when it is to awaken SBs. Braun, Glanzel, and Schubert specifically explored the characteristics of Prince [3]. The phenomenon of the evolution of scientific literature has attracted the attention of researchers as early as the early 1960s, at which time the phenomenon of scientific literature evolution was also termed “Resisted Discovery” [4], “Premature Discovery” [5], and “Delayed Recognition” [6]. From the perspective of citation network, SBs is essentially a distinct phenomenon of the diffusion of scientific knowledge based on citation trajectories, which reveals a crucial mechanism for the diffusion of scientific information through citations [3], and is indispensable for scientific development [7,8]. We refer to the SBs under this perspective as Citation-based Sleeping Beauty (C-SB).

However, the rapid advancement of social media has revolutionized the mode of scientific communication and knowledge diffusion. A multitude of data, such as browsing, saving, and discussion of scientific literature on social media platforms, as well as the quantitative measurement index established by it, have offered a novel perspective and approach to explore the identification and evolution trajectory of SBs and Prince. The knowledge evolution trajectory of scientific literature after publication comprises not only the evolution trajectory based on the citations indicator but also the evolution trajectory based on the social media metrics. We refer to the SBs created by the combination of citations indicator and social media metrics as Altmetrics-based Sleeping Beauty (A-SB). Correspondingly, the all-elements scientific SB from the perspective of comprehensive influence is called Altmetrics-based all elements sleeping beauties (Aa-SB). From this standpoint, how should the Prince of SBs be defined? What type of Prince awakened A-SB? How has the awakening mechanism altered? Of note, reexamining the knowledge diffusion and evolution trajectory of scientific literature from the comprehensive perspective of citations and social media metrics serves a vital supplement and innovative development to the traditional citation-based SBs and Prince research. The contributions of this study are as follows:

1. We define the Prince of SBs from the perspective of Altmetrics and perform an empirical study on the identification of Prince from the standpoint of Altmetrics.
2. We analyze the correlation between A-SB’s Prince and the diffusion trajectory and bibliometric attributes of SBs, followed by exploring the awakening mechanism of A-SB, expanding the research on the awakening mechanism of traditional SBs.

## Literature review

### The evolution trajectory of SBs and prince based on citations

The current research on the evolution of SBs primarily focuses on the citation evolution of SBs, the reasons for the formation and influencing factors of SBs, the identification of Prince, and the awakening mechanism of SBs. Prior studies have defined the “Sleeping–Awakening” time of SBs (Table 1). The Sleeping and Awakening of SBs denote a continuous period, including (1) sleeping period—being cited two times a year on average within 5 years; (2) awakening period—obtain numerous citations (cited >20 times) in a specific period after the sleeping period (>4 years) [1,9–14].

**Table 1:** Definitions of sleeping and awakening in the existing documents of SBs.

Document	Definition of Sleeping	Definition of Awakening
Garfield	In the first ≥5 (>10 years is the best) is low citation, or the frequency of initial citation is low, on average once a year.	Highly Cited Papers
Glanzel et al.	Cited only once within 3 years of initial publication of the paper or up to two times during the first 5 years.	Highly Cited, Cited 100 Times
Glanzel and Garfield	Rarely cited within the first 5 years.	Highly cited within the next 5 years, at least, 50 times; alternatively, 10 times the average impact factors of the journal over a 20-year period
van Raan	Cited at most once per year over a period, or one to two times per year on average.	Cited > 20 times in 4 years after Sleeping
Hou & Yang	PA is less than the duration of Pat, but higher than the critical value of the hysteresis recognition period (Ti)	After Sleeping, PA is higher than Pat, that is, the patent is transferred or it is cited more than 10 times a year

In the citation evolution trajectory, the early long-tailed distribution of citation contributes to the Sleeping of SBs. Reportedly, the long tail can be quantified as the citation delay [15], and its distribution can be used to identify and measure SBs [16,17]. Simultaneously, it can also estimate the formation of SBs to some extent [2]. Particularly, academic development serves a critical factor in the formation of SBs [18]. However, early citation trajectories also merit careful consideration [19]. Although SBs could be highly cited during the later period of diffusion trajectory [13], it is essential to focus on self-citation in late Sleeping [20].

In the citation evolution trajectory, Citations is also the only indicator to study the awakening pattern of SBs. Citations awakens SBs and enables it to arouse considerable scientific attention [21]; these citations are called “Prince” [1]. Thus, the characteristics of Prince [2,3] and identification criteria [22] have become the primary method to explore the awakening mechanism of SBs. Regarding the relationship between Prince and SBs, some scholars believe that SBs embraces its necessity to be awakened [1]. However, some scholars consider that SBs is awakened at random [23]. At the same time, some scholars think that Prince does not necessarily cite SBs directly, but the two are cited together [24]. After this, some scholars believe that the identification of prince should not only analyze the citers of SBs, but also consider the case that the viewpoint of SBs is cited, but SBs is not listed as a reference [25]. The research object of this viewpoint focuses more on the “citation” of Prince than on Prince itself. Recently, van Raan (2015, 2017, 2018) found that SBs is more likely to be cited by patent literature, and the technology drive is the main awakening mechanism of SBs [26–28].

In previous research, the attention on SBs and its awakening mechanism primarily focused on the characteristics and standards of Prince, such as the investigation of the inherent characteristics of the subject content and author relationship of Prince. Owing to the existence of indirect citation and invisible citation, the research on “citation” is overlooked. Besides, the influence of citations during sleeping on the awakening mechanism of SBs and Prince is ignored. Moreover, SBs are cited in some documents during sleeping but have not been successfully awakened. “Heartbeat Spectrum” proposed by Li and Ye observed the frequency of citations during each year of Sleeping [29]; but they focused more on their impact on the formation of SBs. On the other hand, the studies mentioned above examined SBs based on the trajectory of citation evolution. However, with the rapid development of social media platforms, the influence of measuring the impact of scientific literature based on the trajectory of citation evolution has expanded markedly. Besides the citation trajectory of citations, the measurement of the dynamic evolution trajectory should also consider the evolution trajectory of Altmetrics indexes, including Viewed, Saved, Discussed, and Recommended.

### The evolution trajectory of scientific literature based on social media metrics

With the rapid growth of social media, the evolution trajectory of scientific literature comprises not only perspectives based on the evolution trajectory of citations but also evolution trajectories based on social media [30–32]. Altmetrics are the measurement indexes used to measure the social influence diffusion trajectory of scientific literature [33]. Reportedly, the correlation between some Altmetrics and citations is weak [34,35] such as the weak correlation between the number of tweets and citations [36,37]. Several studies have revealed a strong correlation between Altmetrics indexes (Save, Discussion, Download, Read in Mendeley [save], Number of readers in Mendeley, Recommendation measures, The number of tweets, F1000, and Bookmarks) and citations index (Table 2) [38–52]. Nevertheless, Altmetrics indexes and citations index are not simply related, and the differences between different disciplines directly affect their correlation [53]. At present, Altmetrics cannot replace traditional Bibliometrics but has already been a crucial complement of Bibliometrics and Scientometrics [54,55].

**Table 2:** Research on the correlation between Altmetrics indexes and citations index (partial).

Study	Altmetrics indexes	Results
Wardle Bornmann Shema et al.	F1000	Citations of recommended articles in blogs are highly correlated with the Altmetrics indexes
Bornmann and Haunschild	F1000 Prime	Citation-based metrics and readership counts are significantly more related to quality, than tweets
Zahedi et al.	Read in Mendeley [save]	There was a correlation (*r* = 0.49) between the number of readers (preserved) and citations index in Mendeley and the literature with Mendeley readership had a higher citation rate than that without Mendeley readership
Ebrahimy et al.	Save Discussion recommendation measures	The mediating role of visibility and citation relationships in biomedical papers between 2009 and 2013, revealing that these indexes exert a significant impact on the number of future citations to the paper.
Haustein and Siebenlist	Save	Based on 45 journals in the field of physics during 2004–2008, a correlation (*r* = 0.21) was found between Save of the text and the citation.
Shuai et al. Eysenbach Bornmann	microblog counting	Significantly strong correlation between Weibo counts and citations

Accordingly, researchers combined Citations and Altmetrics indexes to explain and define documents of different evolution types, revealing that the performance of highly cited documents on Altmetrics indexes differs from that of citation evolution [56,57]. By combining Bibliometric indexes and Alternative Metrological indexes, a comprehensive document scoring system was constructed [58–61] and was used to conduct empirical research [62–65]. These studies have, to a certain extent, verified the feasibility of comprehensive citations and Altmetrics to measure the influence of academic papers.

Existing studies have focused more on the relationship between Altmetrics indexes and citation, but have not extensively studied or focused on the comprehensive evolution trajectory of scientific literature under the combined action of citations and Altmetrics. Alternatively, the studies have focused on providing a comprehensive scoring system for scientific literature in terms of Altmetrics, in order to evaluate scientifically, but not revealing the specific evolution characteristics of scientific literature under the combined action of Altmetrics indexes and Citations. Recently, Hou & Yang proposed Social media-based Sleeping Beauty and reported the characteristics of SBs recognized on the basis of social media metrics. This study did not incorporate Citations, and the identified SBs embraced no citations [66]. Therefore, there exists certain differences between the evolutionary trajectory and characteristics of SBs under the action of comprehensive indicators combined with social media and citations and the evolutionary trajectory of SBs based on the function of social media indicators. This study preliminarily analyzed the causes of SBs's Awakening on social media platforms, but it did not involve the awakening mechanism in a deep level.

Thus, unlike SBs identified through citation diffusion trajectory of scientific literature [1], we termed the SBs identified through the comprehensive evolution of scientific literature (including Citations and social media indicators) as Altmetrics-based Sleeping Beauty (A-SB). Among them, for all elements SBs under the comprehensive influence trajectory, we termed it Altmetrics-based all elements sleeping beauties (Aa-SB), and Aa-SB is a special A-SB. This study combines Citations and Altmetrics indexes to reidentify SBs (A-SB) and its Prince from the perspective of comprehensive indicators, as well as explore the awakening mechanism of A-SB to complement the research on the awakening mechanism of SBs.

## Data and methods

### Data collection and processing

The literature data (3541 documents) were adopted from the *PLOS Biology* journal since its publication as a sample, and the data were obtained from the *PLOS Biology* Open Access Platform and the Core Collection database of Web of Science. Among them, the data of citations index of the literature were generated from the number of documents published each year by *PLOS Biology*, which is included in the Core Collection database of Web of Science, and the number of citations obtained each year for each article. The social media metrics of the literature include View, Save, Discussed, and Recommended indexes [67–69]. The data were derived from the open-access data of the *PLOS Biology* website; Table 3 shows their specific sources and definition. Among them, Viewed and Discussed are from Open Access Platforms and social networking sites, Saved are from the document management website, Recommended are from scientific paper online recommendation platform, and Citations from the scholarly search engine.

**Table 3:** Definition and source of each index in the journal literature of *PLOS Biology*.

Index	Definition	Data Sources
Citations	Number of times the literature was cited in other academic literature	Web of Science Core Collection
Viewed	Sum of page views and downloads for online document	PLOS, Figshare PubMed Central
Saved	Number of times online documents were saved in the document manager	CiteULike, Mendeley, ORCID
Discussed	Number of times articles have been shared, liked, commented on, and acquired in social tools such as blogs	Nature Blogs, Science Seeker, Research Blogging, Wordpress.com, Twitter, Facebook, Reddit, news media, blogs, reference material, institutions
Recommended	Source data provided by PLOS Publishing Group through online recommendation channels and other platforms for obtaining formal recognition of PLOS research papers	F1000Prime

The indexes are categorized into “Citations,” “Viewed,” “Saved,” “Discussed,” and “Recommended.”

We summarized and cleaned up the relevant data of each article in *PLOS Biology*. Then, we removed the retraction, Correction, Letter, Biographical Item, and other documents, and used Excel and MATLAB 2018b to classify and calculate the selected target data.

We used SPSS Statistics 21 to perform correlation analysis of the indicator information at each stage to measure the correlation between social impact and citations and further determine whether Ab could reflect the comprehensive influence of a document to certify the feasibility of identifying ASB and Prince.

First, the normality test was performed on all indicator data in the sample literature (Table 4), which revealed that they did not conform to the normal distribution data. Thus, the Spearman correlation coefficient was used to analyze the correlation among various indicators. Table 5 shows the correlation coefficient between each indicator and the cited number and Ab.

**Table 4:** The normality test of sample indexes (Shapiro–Wilk).

	Shapiro-Wilk		
	Statistics	df	Sig.
Citations	0.472	3514	0.000
Viewed	0.575	3514	0.000
Saved	0.576	3514	0.000
Discussed	0.135	3514	0.000
Recommended	0.428	3514	0.000
Ab	0.566	3514	0.000

**Table 5:** Correlation analysis of citations and Altmetrics indexes.

Indicators		Citations	Viewed	Saved	Discussed	Recommended	Ab
Citations	Correlation Coefficient	1.000	0.676**	0.748**	0.073**	0.353**	0.741**
	Sig. (double test)	.	0.000	0.000	0.000	0.000	0.000
Viewed	Correlation Coefficient	0.676**	1.000	0.769**	0.198**	0.256**	0.962**
	Sig. (double test)	0.000	.	0.000	0.000	0.000	0.000
Saved	Correlation Coefficient	0.748**	0.769**	1.000	0.164**	0.290**	0.850**
	Sig. (double test)	0.000	0.000	.	0.000	0.000	0.000
Discussed	Correlation Coefficient	0.073**	0.198**	0.164**	1.000	0.031	0.285**
	Sig. (double test)	0.000	0.000	0.000	.	0.071	0.000
Recommended	Correlation Coefficient	0.353**	0.256**	0.290**	0.031	1.000	0.285**
	Sig. (double test)	0.000	0.000	0.000	0.071	.	0.000
Ab	Correlation Coefficient	0.741**	0.962**	0.850**	0.285**	0.285**	1.000
	Sig. (double test)	0.000	0.000	0.000	0.000	0.000	.

The correlation is significant at a confidence level (double test) of 0.05.

The correlation is significant at a confidence level (double test) of 0.01.

As shown in Table, Citations have a significant correlation with Viewed, Saved, Discussed, Recommended, and Ab. The correlation coefficients between Citations, Viewed, Saved, and Ab were all >0.6. Besides, Citations and Discussed, and Citations and Recommended were 0.073 and 0.353, respectively, suggesting a weak correlation. Thus, it is feasible to construct a comprehensive impact evaluation system of Citations and Altmetrics indexes, as well as identify A-SB and its Prince. Moreover, when identifying Prince, we will also focus on Viewed, Saved, and Citations.

### Metrics and methods

We used the Altmetrics-based beauty index (Ab Index) to measure the comprehensive impact generated monthly after the publication of a document, that is, the value of the function of the community of citations index (IA) and social media metrics (IS) as follows:

Ab=f(IA,IS) (1)

To explain the comprehensive evolution trajectory of a document since its publication, we used five types of indicators—Citations (C), Viewed (V), Saved (S), Discussed (D), and Recommended (R)—to elucidate changes of a document in the comprehensive evolution trajectory generated after its publication. These indicators illustrate the influence of a paper from different perspectives. However, what catches our attention is how to objectively assess the comprehensive impact of a document according to these indicators from different perspectives? Thus, we try to deal with these indicators from such a perspective and build a comprehensive trajectory model.

Thus, the dynamic change formula of the **Ab** Index for the *i*-th month since the publication of a document is as follows:

$$Ab_{i}=W_{\mathrm{tv}}\cdot V_{i}+W_{\mathrm{ts}}\cdot S_{i}+W_{\mathrm{td}}\cdot D_{i}+W_{\mathrm{tr}}\cdot R_{i}+W_{\mathrm{tc}}\cdot C_{i}$$ (2)

Among which, W_tv_, W_ts_, W_*td*_, W_tr_ and W_tc_ are the corresponding weights of V, S, D, R and C respectively; *i* denotes time, which in this study is the *i*-th month after the publication of a document; and *Ab*_i_ denotes the comprehensive influence of the *i*-th month after the document’s publication. It should be noted that Web of Science does not provide the monthly citation of all literature, only the citation data of literature in each year can be obtained, so the Ci here is the average of the monthly citation quantity of literature in a certain year [70]. For example, a document is cited 12 times in the third year after publication, suggesting that each month in the third year is cited once.

For the determination of weightsW_tv_W_ts_W_*td*_W_tr_W_tc_ *W*_tv_, *W*_ts_, *W*_td_, *W*_tr_, *W*_tc_, using the Analytic Hierarchy Process, we constructed a structural matrix, based on the effect of five types of indicators on the comprehensive evolution trajectory of a document and assigned different weight values to each indicator. Among these, we selected the value in the model matrix based on 9 scale, which can fulfill the needs of ranking the five indicators under a single rule. Furthermore, by using the MATLAB tool, we accurately evaluated the five indexes and obtained a comprehensive weight.

The basic steps of the analytic hierarchy process are as follows:

Compare the influence of n factors *X*_1_,*X*_2_,…,*X*_*n*_ on a factor (influence) at the previous level, you can choose *X*_i_ and *X*_j_ from *X*_1_,*X*_2_,…,*X*_*n*_ to compare their contribution to influence (Or importance). Assign values to *X*_i_/*X*_j_ according to the following “1–9 scale.”

**Table:** 

**Xij**	**Implication**
1	The influence of *X*_i_ is equivalent to that of *X*_j_
3	The influence of *X*_i_ is slightly stronger than that of *X*_j_
5	The influence of *X*_i_ is stronger than that of *X*_j_
7	The influence of *X*_i_ is obviously stronger than that of *X*_j_
9	The influence of *X*_i_ is absolutely stronger than that of *X*_j_
2,4,6,8	The ratio of influence of *X*_i_ and *X*_j_ is between the above two adjacent levels
1,1/2,…,1/9	The ratio of influence of *X*_i_ and *X*_j_ is the inverse of the ratio above

Building the model matrix:

**Table:** 

**Xij**	**Vi**	**Sa**	**Re**	**Di**	**Cd**
**Vi**	1	1/2	1/7	1/7	1/9
**Sa**	2	1	1/5	1/5	1/8
**Re**	7	5	1	1	1/5
**Di**	7	5	1	1	1/5
**Cd**	9	8	5	5	1

$$B=\left[\begin{array}{ccccc} 1 & 1/2 & 1/7 & 1/7 & 1/9\\ 2 & 1 & 1/5 & 1/5 & 1/8\\ 7 & 5 & 1 & 1 & 1/5\\ 7 & 5 & 1 & 1 & 1/5\\ 9 & 8 & 5 & 5 & 1 \end{array}\right]$$

By evaluating, the maximum of its eigenvalues is *λ*_max_ = 5.2837, and the eigenvector at the maximum eigenvalue is as follows:

$${\left(0.0515\;0.0770\;0.2827\;0.2827\;0.9919\right)}^{T}$$

By performing consistency test on the results, $CI=\frac{\lambda \max‐n}{n‐1}=\frac{5.2837‐5}{5‐1}=0.0709$, the consistency ratio was $\mathrm{CR}=\frac{\mathrm{CI}}{\mathrm{RI}}=0.063<0.1$. Thus, RI is random consistency test, when *n* = 5, RI = 1.12. Hence, the matrix passes the consistency test. By conducting the Normalization Processing on the eigenvector, the weights were obtained, that is, *W*_tv_ = 0.0321, *W*_ts_ = 0.048, *W*_td_ = 0.176, *W*_tr_ = 0.176, and *W*_tc_ = 0.5679. Hence, the Ab Index can be expressed as follows:

$$Ab_{i}=0.0321V_{i}+0.048S_{i}+0.176D_{i}+0.176R_{i}+0.5679C_{i}$$ (3)

From a citation-based perspective, the time-statistical unit used by the C-SB Institute is based on years. However, since the publication cycle of several documents is monthly, it creates a large time gap between the documents published in January and December each year when the statistics are collected on an annual basis. If a document published in January awakens after 4 years of Sleeping, then its Sleeping time is ≥47 months; however, if a document published in December awakens after 4 years of Sleeping, its Sleeping time is ≥36 months.

Nonetheless, from the perspective of Altmetrics-based, the spread of literature on social media platforms is faster, and the diffusion trajectory of the literature in monthly units can more precisely denote the dynamic diffusion process of the literature. Thus, under the comprehensive evolution trajectory, we adopted Th = 36 months as the standard, that is, when a document awakens after Sleeping for ≥36 months, it was considered to be Altmetrics-based SB (Delayed Recognition).

Hence, the Combined Ab index, we redefined the Sleeping and Awakening of Altmetrics-based SB.

**Awakening**: In the C-SB identification research, it typically takes years as the unit of time. Scientific literature needs to be awakened for 4 consecutive years before it can be termed as true Awakening; however, it is not necessarily continuous in terms of monthly time. To ensure the research consistency, we selected four consecutive time intervals to determine the literature as Awakening. However, under the social media platform, we define SBs enter the Awakening stage the comprehensive influence value for 4 consecutive months (Ab_**i**_) is higher than the average monthly comprehensive influence of all the literature of the journal $(\bar{Ab).}$ We selected the indicator because in the study of the Awakening of SBs, it is unfair to compare publications from different scientific disciplines, as significant difference exists in speed and frequency of citation accumulation across different fields of science [70–72].

**Sleeping**: The average comprehensive influence of the literature for 4 consecutive months is $\leq \bar{Ab}/2$ (${Ab}_{△n}\leq \bar{Ab}/2,$ Sleeping). Before entering the Awakening, we consider that SBs is in the Sleeping stage, which includes both Sleeping and Dogsleep.

**Dogsleep**: In a specific period, when the Awakening or Sleeping of a document does not change continuously (continuous Awakening or Sleeping does not exceed 4 months), the document is considered to have entered Dogsleep. Precisely, we defined that when $\bar{Ab}/2<{\bar{Ab}}_{\left(j-i\right)}<\bar{Ab}$, or when the comprehensive influence value (**Ab**_**i**_) is not greater than the average value of the comprehensive influence of all literature ($\bar{Ab}$) (${\bar{Ab}}_{\left(j-i\right)}>\bar{Ab}$) in the journal for four consecutive months, the SBs is in Dogsleep.

**Awakening time** (Tw-k): The duration of Awakening, where *k* denotes the times Awakening, and when *k* = 1, Awakening time.

**Dogsleep time** (Tb-k): The duration of Dogsleep, where *k* denotes the times of continuous Dogsleep, and when *k* = 1, it denotes the first Dogsleep time.

**Sleeping time** (Ts-k): The Sleeping duration, where *k* denotes the times of continuous Sleeping, and when *k* = 1, it denotes the first Sleeping time.

**Awakening intensity:** To describe the literature characteristics at different stages, we used two variables, average (${\bar{Ab}}_{\left(j-i\right)}$) and standard deviation (σ). We assumed that the process of the Sleeping and Awakening is as follows: Sleeping/Dogsleep–Awakening–Sleeping/Dogsleep–Awakening–Sleeping/Dogsleep. When in Sleeping, ${\bar{Ab}}_{\left(j-i\right)}\leq \bar{Ab}/2$, the smaller the ${\bar{Ab}}_{\left(j-i\right)}$ and σ, the stronger the Sleeping intensity of the literature. Conversely, when in Awakening, ${\bar{Ab}}_{\left(j-i\right)}\geq \bar{Ab}$, the larger the ${\bar{Ab}}_{\left(j-i\right)}$ and σ, the stronger the Awakening intensity of the literature. Likewise, during Dogsleep, the larger the ${\bar{Ab}}_{\left(j-i\right)}$ and σ, the stronger the Dogsleep intensity of the literature.

In this study, we used the following definitions (Table 6) of the degree of continuous change in different states as the criteria for identifying A-SB and AA-SB.

**Table 6:** Identification methods of Altmetrics-based SB.

Document type	First Sleeping/Dogsleep		First Awakening		Second Sleeping/Dogsleep		Second Awakening	
	time	Ab (j-i)	time	Ab (j-i)	time	Ab (j-i)	time	Ab (j-i)
A-SB	Ts-1≥Th	≤ $\bar{A}b$ /2	Tw-1≥4	≥Ab	uncertainty	——	——	——
AA-SB	Ts-1 <Th or Tb-1 <Th	≥0	Tw-1≥4	≥Ab	Ts-2≥Th	≤ $\bar{A}b$/2	Tw-2≥4	≥Ab

Besides, to avoid the ambiguity of expression, we need to define some concepts of “Dogsleep” and “Prince.”

**Dogsleep**: In the trajectory study of C-SB, scholars focused on the trajectory characteristics of Sleeping-Awakening. However, some scholars have noticed the low citation [29] and temporarily high citation of SBs during Sleeping [73]. Under this phenomenon, there is no continuous change in the Awakening or Sleeping of a document, which is known as entering Dogsleep.

**Prince:** The literature that awakens SBs under the citation track is called Prince. Under the social media platform, Citations is no longer the only indicator for awakening SBs. Citation a specific academic behavior. Under the social media platform, the Awakening of A-SB is achieved by the combined action of various indicators, but there must be a decisive indicator with the largest proportion, which reflects the most prominent academic or social behaviors that awaken and maintain A-SB's Awakening, and its appearance makes A-SB highly concerned after a long period of Sleeping. Specifically, we define that Prince needs to meet the following requirements: ①It will have the highest proportion of overall influence in the first month of the Awakening stage, thus awakening A-SB. ②It is expected to have a sustained high influence during the Awakening stage, to contribute most to maintaining the Awakening of A-SB.

“Prince” is not defined as the leading indicator in the first month of the Awakening stage because on social media platforms, the real Awakening of A-SB is a continuous process, not an instant; this is important which criteria distinguishes Awakening from Dogsleep. In addition, in the current academic communication environment, the definition of “Prince” only as the first citer is not conducive to considering the motivation of Awakening and accurately defining the Sleeping-Awakening stage [22].

## Results

We used the methods and definitions mentioned above to conduct empirical research on the retrieved *PLOS Biology* collection. After identifying the A-SB, we examined Prince of A-SB, and explored the awakening mechanism of A-SB, supplementing the research of traditional SBs.

### Identification of A-SB

**Fig 1:**
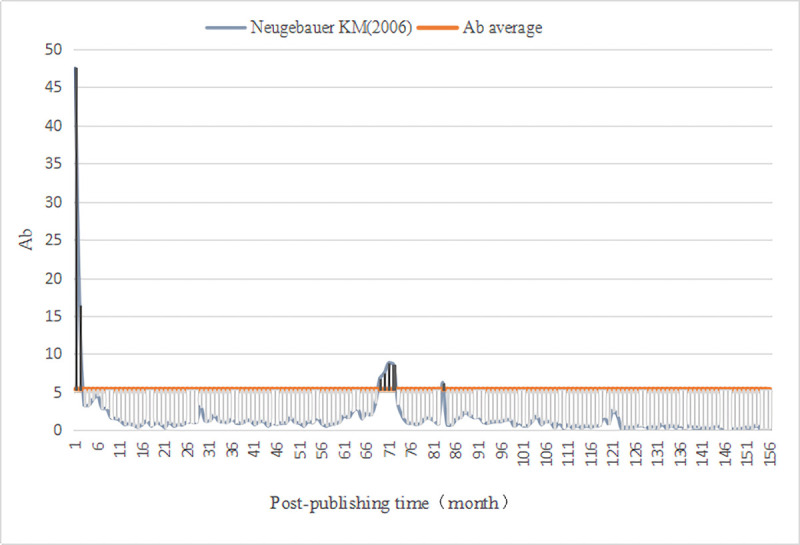
Ab Trajectory of Neugebauer KM (2006).

**Fig 2:**
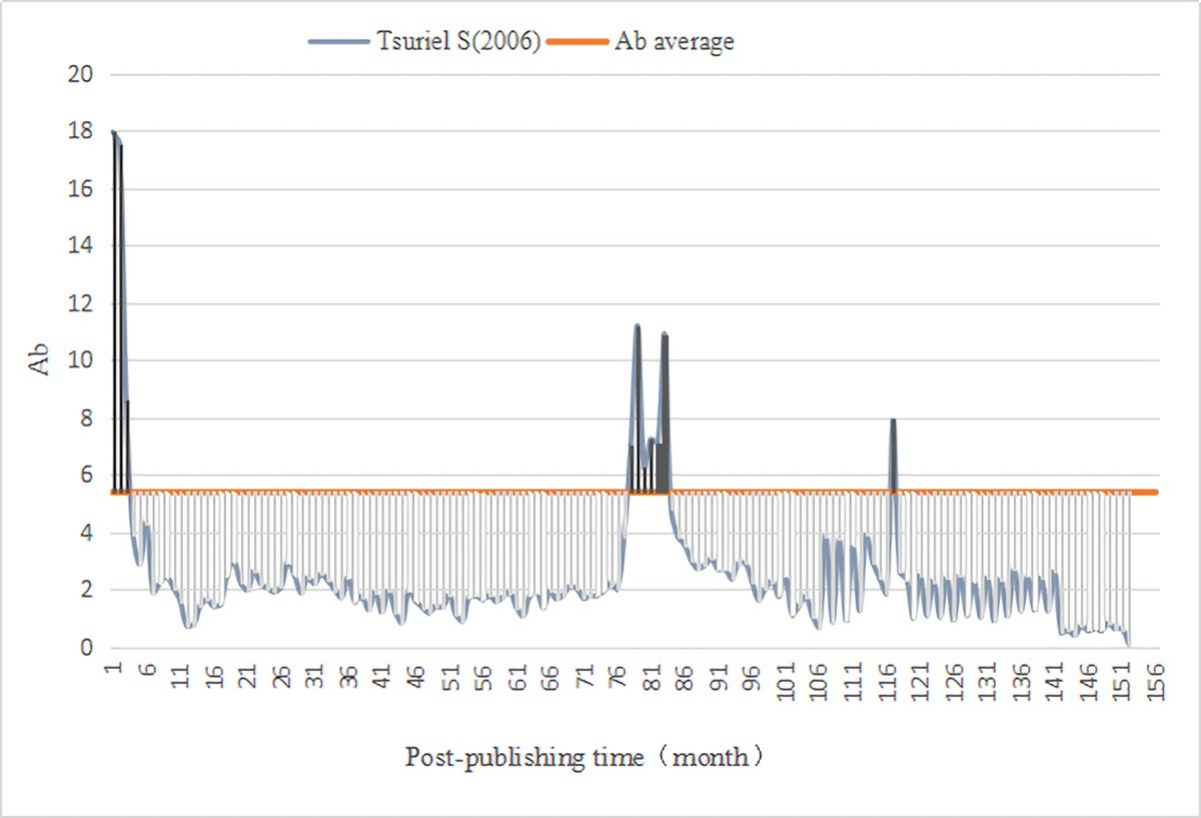
Ab Trajectory of Tsuriel S (2006).

**Fig 3:**
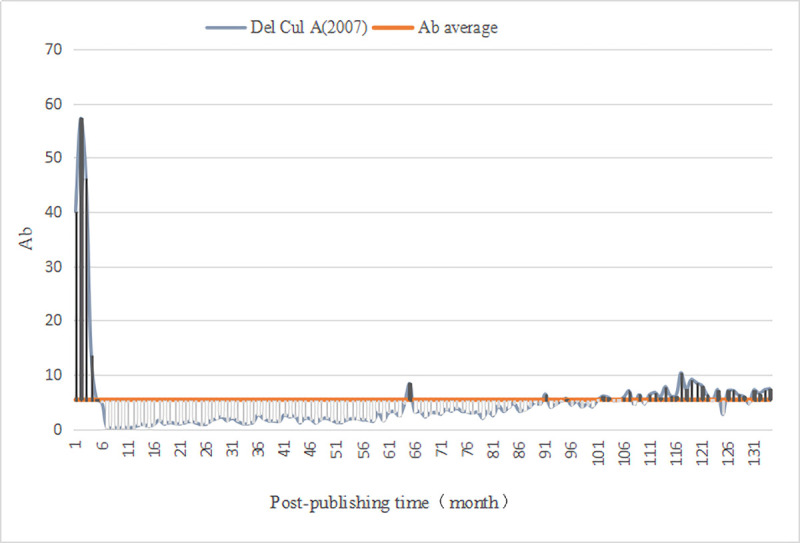
Ab Trajectory of Del Cul A (2007).

**Fig 4:**
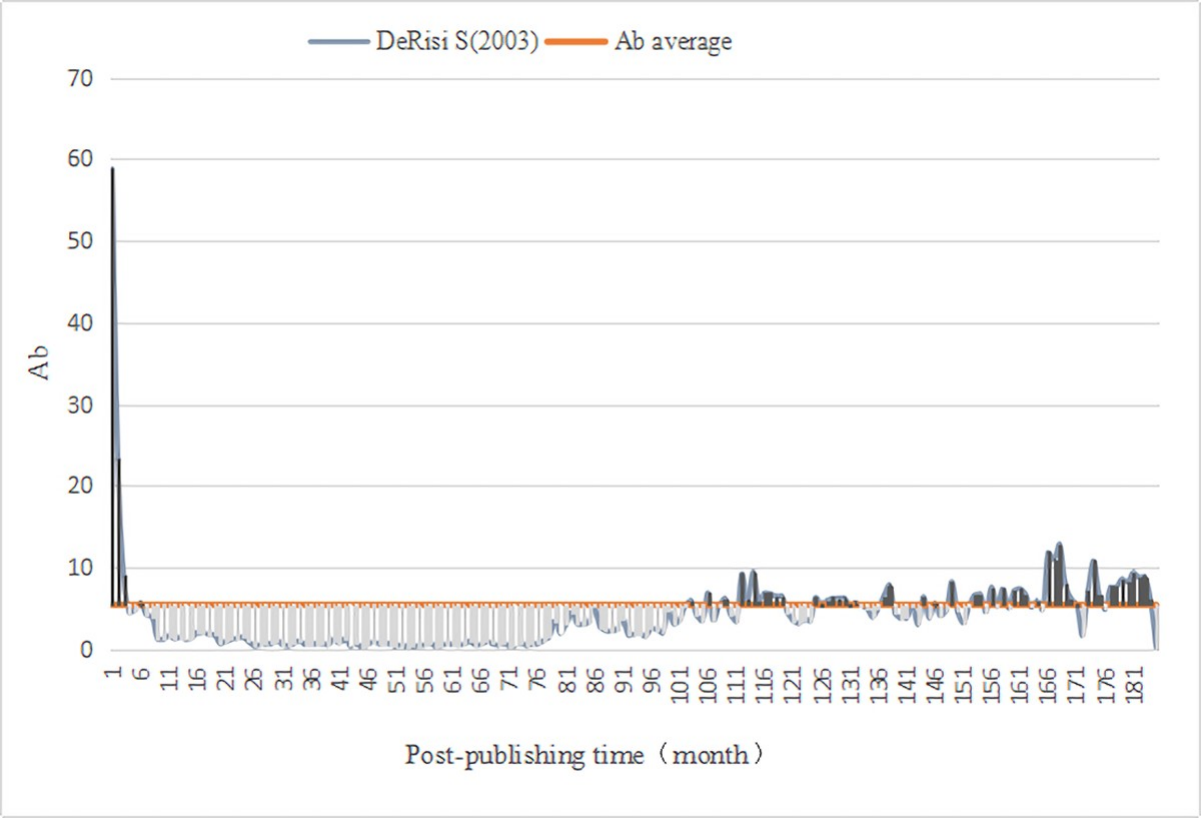
Ab Trajectory of DeRisi S (2003).

**Fig 5:**
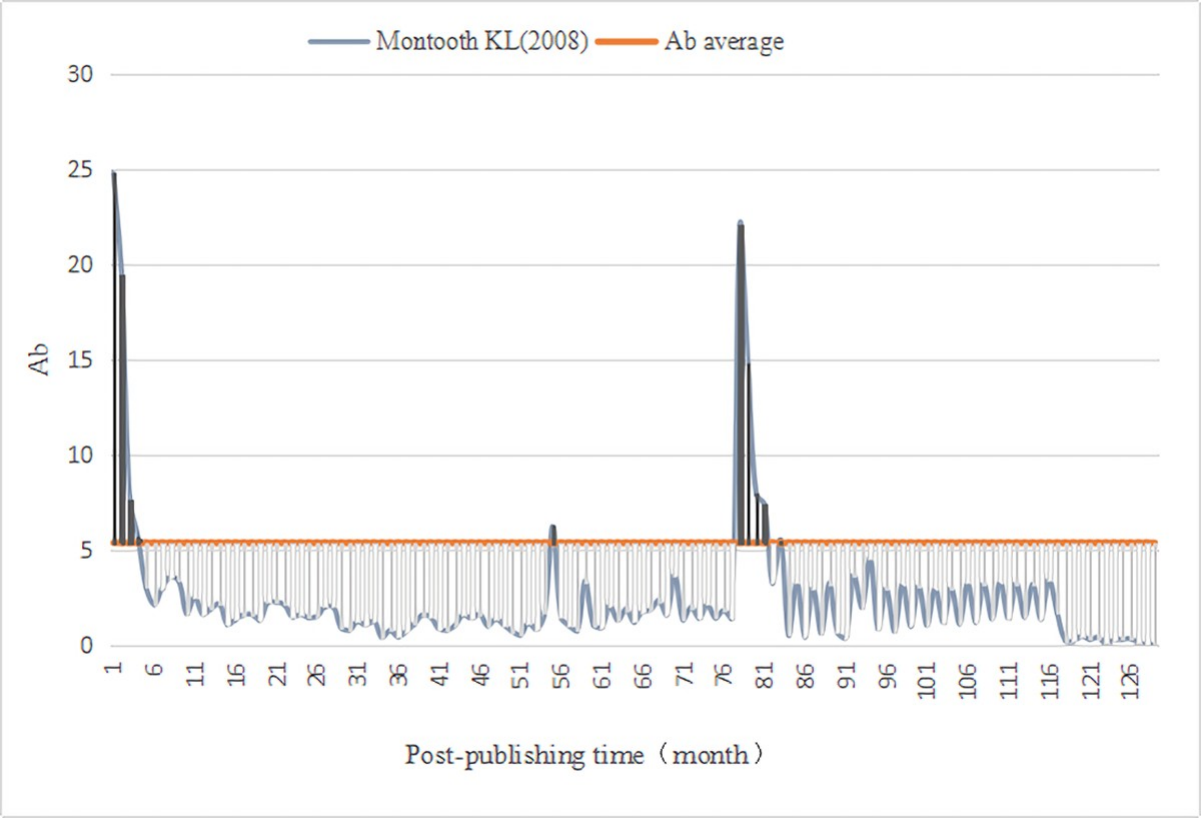
Ab Trajectory of Montooth KL (2008).

**Fig 6:**
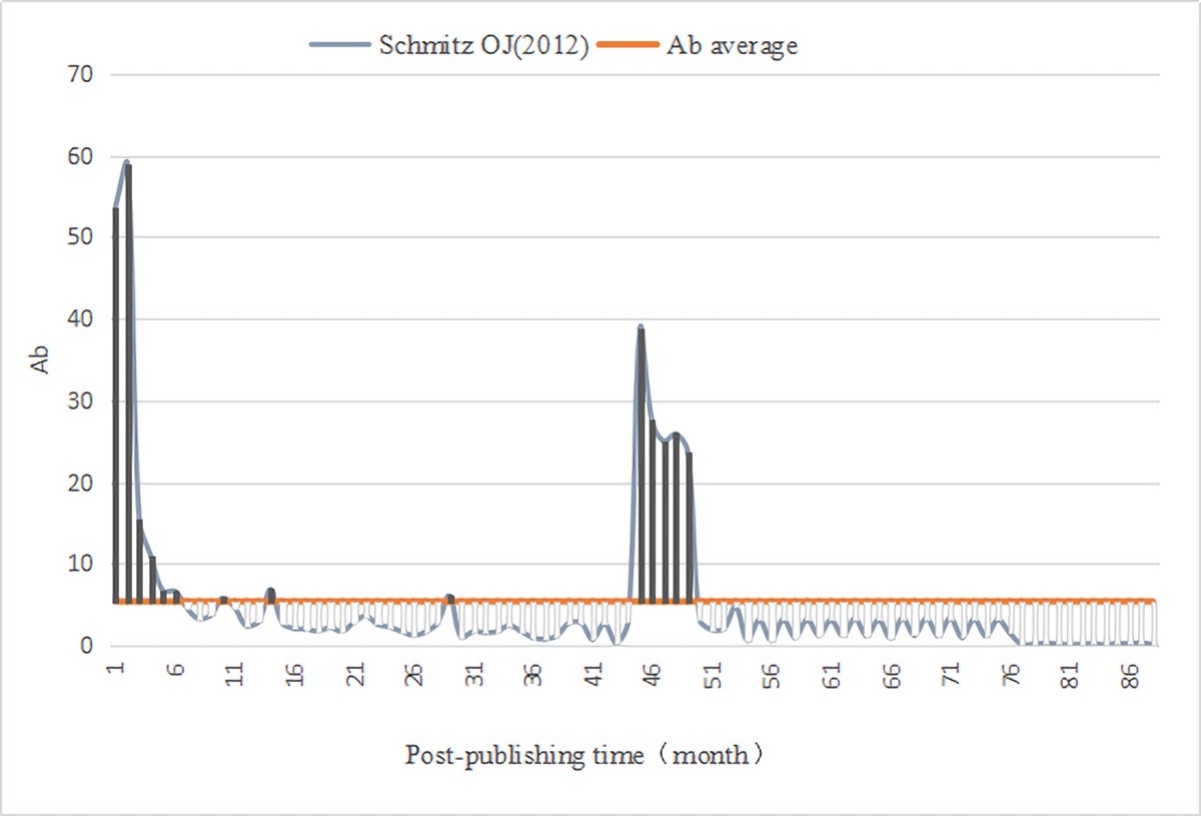
Ab Trajectory of Schmitz OJ (2012).

**Fig 7:**
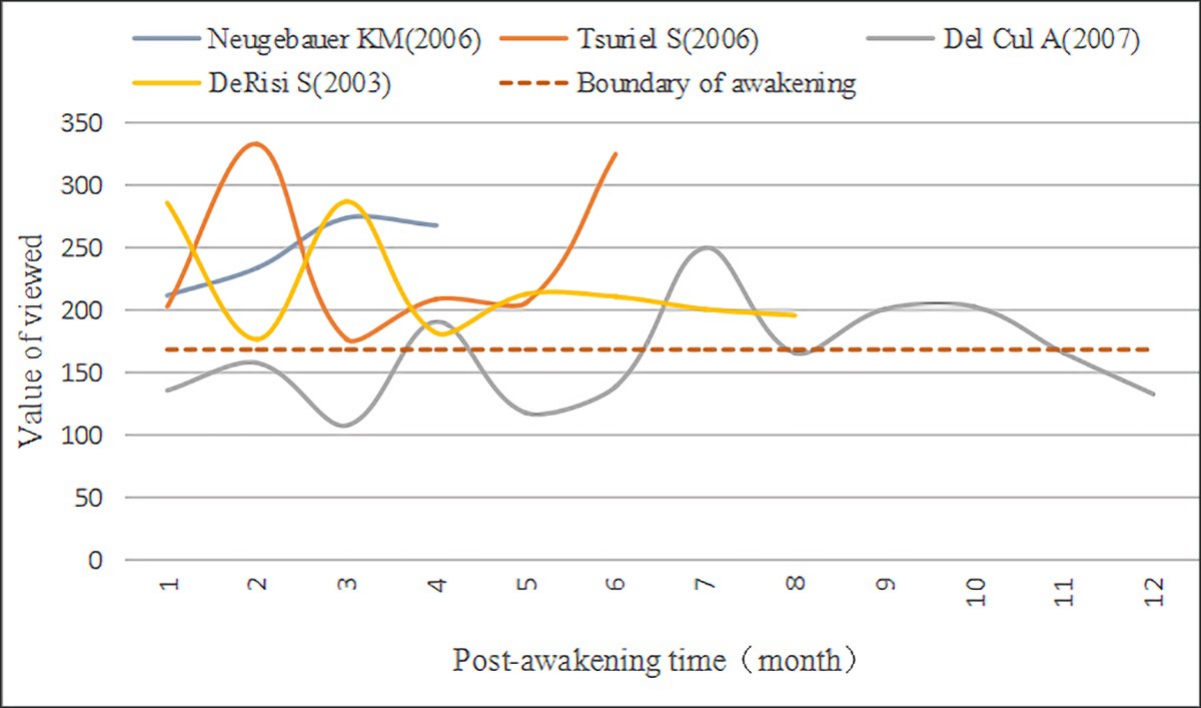
Time distribution of viewed of A-SB in the awakening stage. The Boundary of awakening is formed by the influence of Viewed solely, in order to check whether the documents could enter awakening only by Viewed.

We evaluated the monthly comprehensive influence of all documents in *PLOS Biology*. First, we calculated the average value ($\bar{Ab}=5.38$) of the comprehensive influence. Thus, for the literature published in *PLOS Biology*, the Awakening is defined as (Ab_*n*_⋯Ab_n−3_)>5.38 and the Sleeping is Ab_△*n*_≤2.69. Then, we computed the monthly comprehensive influence value of 3541 documents since publication, as well as identified A-SB and Aa-SB in the sample (Table 7) based on the degree of continuous change in their impact value, discovering 4 A-SB (Figs 1–4) and 7 Aa-SB (Figs 5 and 6) [74–86]. We found that the trajectories of A-SB and Aa-SB are right skewed of long tail, and their Ab presented a high peak in the early stage, and then entered Dogsleep. After being awakened by Prince, they obtained a sustained high Ab. The difference was that the duration of the high Ab of A-SB did not exceed the four consecutive months defined by Awakening, and the duration of Aa-SB was no less than four months. Therefore, we considered that A-SB was in Dogsleep and was not awakened, while early Awakening occurred in Aa-SB. It should be noted that Aa-SB is a special form of A-SB. The time range we studied was the Delayed Recognition stage of A-SB, during which stage Aa-SB occurred early Awakening and second Awakening.

**Table 7:** A-SB in *PLOS Biology*.

Type	Document	DOI	Citations	Ab	Document type
A-SB	Neugebauer KM (2006)	10.1371/journal.pbio.0040097	6	270.2223	Editorial Material
	Tsuriel S (2006)	10.1371/journal.pbio.0040271	107	427.0537	Article
	Del Cul A (2007)	10.1371/journal.pbio.0050260	231	602.3114	Article
	DeRisi S (2003)	10.1371/journal.pbio.0000057	4	735.8933	Editorial Material
Aa-SB	Montooth KL (2008)	10.1371/journal.pbio.0060213	25	325.3092	Editorial Material
	Khalturin K (2008)	10.1371/journal.pbio.0060278	51	453.0254	Article
	Schmitz OJ (2012)	10.1371/journal.pbio.1001248	6	454.3298	Editorial Material
	MacDonald PE (2006)	10.1371/journal.pbio.0040049	35	571.1707	Editorial Material
	Powell K (2007)	10.1371/journal.pbio.0050338	13	477.5412	Editorial Material
	Alerstam T (2007)	10.1371/journal.pbio.0050197	125	435.3883	Article
	Pan J (2007)	10.1371/journal.pbio.0050333	9	502.6319	Editorial Material

### The identification of prince of A-SB

According to the definition of Prince in 3.2, we identified the Prince of A-SB.

#### Analysis of the proportion of various indicators in the awakening stage of A-SB

We analyzed the proportion of each indicator of Ab in the Awakening stage of 11 A-SB. Given that 11 A-SB have not been affected by Recommended during Awakening, we will not take Recommended into consideration in the following analysis. As shown in Table 8, we found that the indicator of the largest proportion of Ab in 11 A-SB was Viewed. Among the average values of the proportions, the largest is Viewed and the smallest is Discussed.

**Table 8:** Proportion of various indicators of A-SB in the awakening stage.

Document	Citations	Saved	Discussed	Viewed	Recommended
Neugebauer KM(2006)	0.00%	0.00%	0.00%	100.00%	0.00%
Tsuriel S(2006)	6.74%	0.00%	1.4%	91.86%	0.00%
Del Cul A(2007)	18.11%	5.65%	0.65%	75.59%	0.00%
DeRisi S(2003)	0.00%	0.00%	1.24%	98.76%	0.00%
Montooth KL(2008)	1.53%	0.43%	0.00%	98.04%	0.00%
Khalturin K(2008)	1.06%	2.00%	0.43%	96.51%	0.00%
Schmitz OJ(2012)	0.19%	2.21%	0.00%	97.60%	0.00%
MacDonald PE(2006)	1.23%	0.89%	0.15%	97.73%	0.00%
Powell K(2007)	0.29%	1.19%	0.00%	98.52%	0.00%
Alerstam T(2007)	1.45%	0.00%	0.22%	98.33%	0.00%
Pan J(2007)	0.00%	0.00%	0.71%	99.29%	0.00%
Mean	2.78%	1.12%	0.44%	95.66%	0.00%

The proportion values in the table are derived from the average percentage of the indexes in AB value in the awakening stage. For example, in Pan (2007), Viewed accounted for 99.29% of *AB*.

In order to observe the changing trend of various indicators in different stages of A-SB trajectory, we summarized the proportion of the comprehensive influence of various indicators in different stages of A-SB in Delayed Recognition stage (Table 9). It can be found that the proportion of Viewed occupied the highest proportion in the overall influence of the Delayed Recognition stage, and that the proportion of it after A-SB is awakened is greater than that of A-SB in sleeping. Differently, the proportion of other indicators decreases after A-SB awakens. Among which, the decline of Citations is the most pronounced, and it works when A-SB is in Sleeping, but not strong enough to awaken A-SB. Only Viewed's influence soars before and after A-SB's awakening.

**Table 9:** The changes of influence proportion of the indicators of Altmetrics in the delayed recognition stage of A-SB and Aa-SB.

Indicator Stage	Dogsleep	Sleeping	Awakening	Delayed Recognition
Viewed	90.7%	89.9%	95.7%	91.4%
Saved	0.4%	1.8%	1.1%	1.5%
Discussed	1.1%	1.3%	1.2%	0.9%
Recommended	0	0	0	0
Citations	7.7%	7%	2%	6.2%

The proportion values in the table are derived from the average percentage of the indexes in *AB* value in a certain stage. For example, in the sleeping stage, Viewed accounted for 89.9% of *AB*.

Therefore, according to the proportion of the comprehensive influence in the Awakening stage, Viewed is the most active indicator in awakening 11 A-SB, while Saved and Citations play an essential role in some A-SB. However, the proportion of indicators cannot explain the real function of them in awakening A-SB, because there may be uneven or relatively dispersed distribution of indicators. In order to determine whether these indicators really awaken A-SB, it needs to be analyzed in combination with their changing trend and specific trajectory before and after awakening.

#### Trajectory analysis of each indicator in the awakening stageof A-SB

In order to explore the specific role of each indicator in Awakening of A-SB and find out the reason of A-SB's awakening, we presented the influence track of each indicator of A-SB in the form of stacked rectangle graph and analyzed them (Table 10). Among them, in order to compare the causes of two Awakening of AA-SB more clearly, the early Awakening of AA-SB is displayed on the same track as the second Awakening.

**Table 10:** Trajectory analysis results of A-SB in awakening.

Document	Duration of Awakening (months)	Ab of Awakening	The indicator with the largest proportion	The indicator for awakening	The indicator for maintaining
Neugebauer (2006)	4	31.5864	Viewed	Viewed	Viewed
Tsuriel S (2006)	6	50.560	Viewed	Viewed	Viewed
Del Cul (2007)	12	87.6327	Viewed	Viewed Citations	Viewed Citations Saved
DeRisi (2003)	8	56.7185	Viewed	Viewed	Viewed
Montooth (2008)	8	111.3879	Viewed	Viewed	Viewed
Khalturin (2008)	9	161.084	Viewed	Viewed	Viewed Saved
Schmitz (2012)	11	293.1552	Viewed	Viewed	Viewed
MacDonald (2006)	16	231.2074	Viewed	Viewed	Viewed
Powell (2007)	11	197.9274	Viewed	Viewed	Viewed Saved
Alerstam (2007)	8	78.3839	Viewed	Viewed	Viewed Citations
Pan (2007)	9	124.4971	Viewed	Viewed	Viewed

The recognition results of the indexes in the table come from the calculation and description of the track (see Supporting information for specific pictures).

Among 4 A-SB, except Del Cul A(2007), A-SB can be awakened and maintain its awakening only by Viewed. Based on the track of Del Cul A(2007), we found that during the first, second, third, fifth, sixth and twelfth months of its Awakening, Viewed alone can not awaken A-SB, but requires cooperation with Citations and Saved. However, because Saved has less influence, it is far from being able to awaken the literature without Viewed. Citations has a certain influence, but due to the dispersion of its distribution, its monthly influence is not strong enough to dominate the Awakening of A-SB. Viewed's influence is close to A-SB's Awakening boundary and enjoys a continuity, so we believe that it is Viewed that dominates the awakening of Del Cul A(2007). This suggests that, from the perspective of comprehensive influence, academic researchers' citation of literature is still one of the reasons to awaken A-SB, but it is no longer the most significant factor. A-SB is also subject to mentions, discussions, sharing and other communication behaviors by users on social networking websites in the Awakening stage, but from the perspective of the influence of Discussed, it is not the reason for A-SB's awakening.

In 7 Aa-SB, we found that the indicator that contributes to their awakening was Viewed. In the second Awakening, Khalturin K (2008) and Powell K (2007) were awakened by Viewed alone, and Saved participated in the maintenance of Awakening; Alerstam T (2007) was also awakened by Viewed alone, but Citations participated in maintaining Awakening. However, because the influence of Viewed was near the Awakening boundary, the influence of Saved and Citations was minimal, and their role was more like assisting Viewed's awakening. The second Awakening of other Aa-SB was completely dominated by Viewed alone.

**Fig 8:**
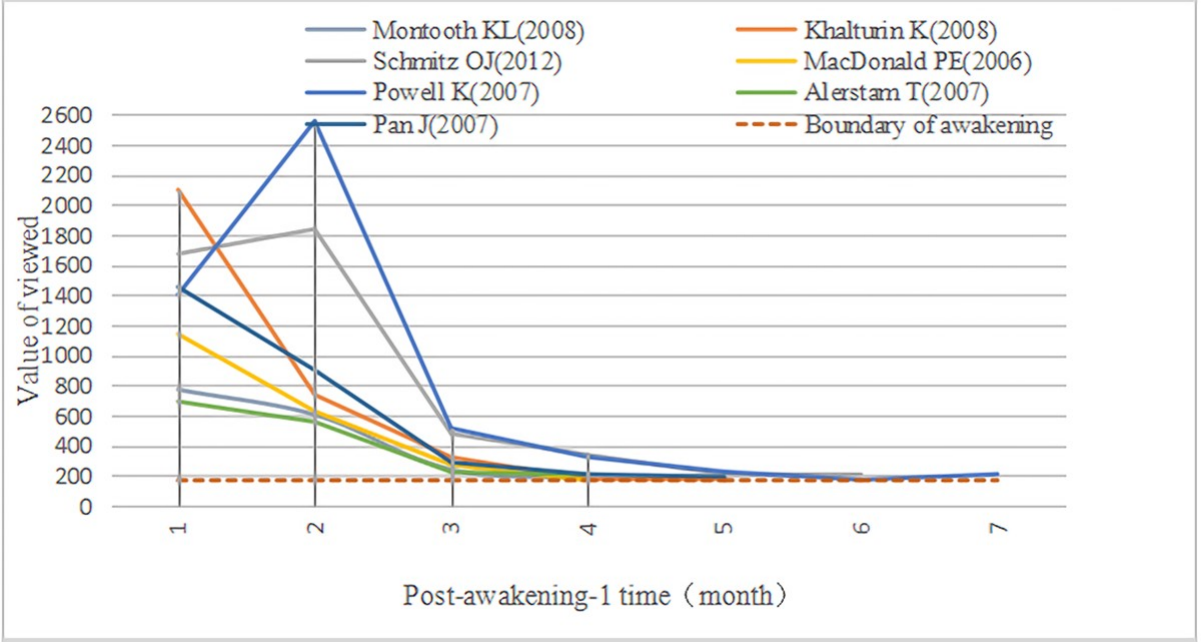
Time distribution of viewed of Aa-SB in early awakening. The Boundary of awakening is formed by the influence of Viewed solely, in order to check whether the documents could enter awakening only by Viewed.

**Fig 9:**
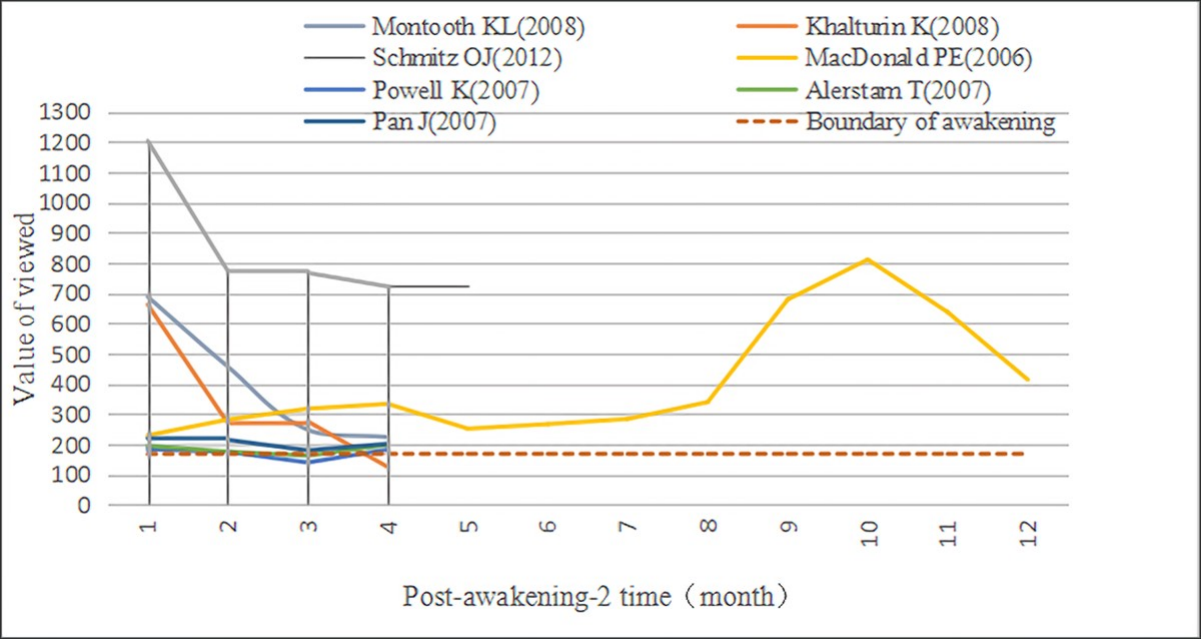
Time distribution of viewed of Aa-SB in second awakening. The Boundary of awakening is formed by the influence of Viewed solely, in order to check whether the documents could enter awakening only by Viewed.

Although the leading inicators of A-SB and Aa-SB's early and second Awakening are Viewed, their trajectory and influence are different (Figs 7–9). We found that Viewed’s distribution curve of A-SB in the Awakening stage showed a slight vibration trend, and the whole was approaching the Awakening boundary line. Viewed’s distribution curve of Aa-SB showed a decreasing trend in the early Awakening, but Viewed showed no evident trend in the second Awakening, but all of them were near the Awakening boundary line. After separating the first and second Awakening of Aa-SB, we observed that the Viewed obtained during the early Awakening of Aa-SB was significantly greater than that of the second Awakening, and the Viewed of the second Awakening was generally greater than that of A-SB’s Awakening. In addition, the Discussed, Saved, and Citations of Aa-SB all focused on the second Awakening, which indicates that Aa-SB was highly viewed and downloaded by users shortly after publication, but then quickly lost the user's attention until being awakened by browsing and downloading again, it began to be widely discussed, saved and cited by website users and academic researchers.

Therefore, considering the reason why 4 A-SBs and 7 Aa-SBs were awakened, we believe that Prince of 11 A-SB are Viewed, and their awakening is all dominated by the browsing and downloading of the users’ social behavior. Moreover, compared with normal A-SB, Aa-SB is more sensitive to Prince’s “kiss.” Thanks to Viewed, their overall influence in final Awakening is higher than that of A-SB. In addition, 11 documents have not been officially recognized and recommended by academic researchers during the Awakening stage. Citations have played a role in the awakening of some documents, but it is no longer the most important indicator of A-SB’s Awakening. However, because Saved and Citations have a certain proportion in the Awakening stage, although Viewed is Prince, some documents cannot maintain the Awakening of A-SB by Viewed solely, which makes their reason for awakening more complicated.

### Research on the awakening mechanism of A-SB

Based on the results of the normality test in Section 3.1, we used the Spearman correlation coefficient to complete the following analysis.

#### Correlation analysis of the indicators of A-SB in awakening

In the identification of Prince, we found that there were two awakening methods of A-SB: one is that Prince solely awakens A-SB; the other is that Prince awakens A-SB in corporation with other indicators. In order to further analyze the interaction mechanism of Prince and other indicators during the awakening process of A-SB, we conducted a Spearman correlation analysis on the identified Prince of 11 A-SB, Viewed, and other indicators in Awakening (Table 11). This indicates that in the Awakening of A-SB, the massive browsing of users, on one hand, can awaken A-SB solely, and on the other hand, can guide users’ saving behaviour, thereby assisting the awakening of Prince or improving the comprehensive influence of Awakening. In this interesting phenomenon, the relationship between Viewed and Saved resembles a prince and a follower, Saved accompanies Viewed and assists it to awaken A-SB.

**Table 11:** Correlation analysis of indicators in awakening.

Indicators		Citations	Saved	Discussed	Viewed	Ab
Citations	Correlation Coefficient	1.000	0.396	0.083	0.046	0.092
	Sig. (double test)	.	0.227	0.808	0.893	0.788
Saved	Correlation Coefficient	0.396	1.000	−0.305	0.658*	0.725*
	Sig. (double test)	0.227	.	0.361	0.028	0.012
Discussed	Correlation Coefficient	0.083	−0.305	1.000	−0.202	−0.183
	Sig. (double test)	0.808	0.361	.	0.551	0.590
Viewed	Correlation Coefficient	0.046	0.658*	−0.202	1.000	0.991**
	Sig. (double test)	0.893	0.028	0.551	.	0.000
Average Ab	Correlation Coefficient	0.092	0.725*	−0.183	0.991**	1.000
	Sig. (double test)	0.788	0.012	0.590	0.000	.

The correlation is significant at a confidence level (double test) of 0.05.

The correlation is significant at a confidence level (double test) of 0.01.

In addition, there is no correlation between citations, Altmetrics indexes and Ab, because of the discontinuity of the distribution of citations in Awakening. This demonstrates that the role of citations in the Awakening of A-SB is not related to Altmetrics indexes. It reflects the academic influence of A-SB and is relatively independent in the process of awakening A-SB. However, citations have played an essential role in awakening some A-SB. From the perspective of comprehensive influence, we still need to pay attention to traditional Citations.

Therefore, based on the above findings, we believe that there is a deeper internal mechanism in the awakening mechanism in which Prince cooperates with other indicators to awaken A-SB. The indicators, including Prince, are not completely independent of each other when they play a role in Awakening, reflecting that the communication between different social behaviors under the social media platform makes the awakening mechanism of SBs more diverse.

#### Correlation analysis of prince and indicator information at each stage

The trajectory of A-SB in Delayed Recognition on the social media platform is dynamic and continuous. In order to further study whether Prince is related to other stages of A-SB, and explore the awakening mechanism of A-SB from a holistic perspective, we conducted correlation analysis between Prince of A-SB, Awakening intensity, the intensity of other stages and Viewed (Table 12). Among them, we integrated the data of Aa-SB's two awakenings to ensure the consistency of A-SB and Aa-SB research.

**Table 12:** Indicator information of A-SB and Aa-SB.

Sleeping Beauty Type	Document name	Status/stage	Time	Average Ab	Standard deviation of Ab	Viewed
A-SB	Neugebauer KM (2006)	Dogsleep	11	8.542	12.877	2927
		Sleeping stage	68	2.276	34.592	4822
		Awakening stage	4	7.796	0.920	984
	Tsuriel S (2006)	Dogsleep	11	6.526	5.545	2026
		Sleeping stage	77	2.447	7.379	4698
		Awakening stage	6	8.278	2.165	1446
	Del Cul A (2007)	Dogsleep	47	4.013	1.367	4036
		Sleeping stage	110	2.584	3.221	5587
		Awakening stage	12	7.344	1.407	1920
	DeRisi S (2003)	Dogsleep	30	6.821	10.301	6293
		Sleeping stage	111	2.551	36.002	8699
		Awakening stage	8	7.090	1.377	1745
AA-SB	Montooth KL (2008)	Early Awakening	4	14.494	9.197	1789
		Dogsleep	6	3.848	1.169	706
		Sleeping stage	73	1.742	0.904	3597
		Second Awakening	4	13.230	6.885	1613
	Khalturin K (2008)	Early Awakening	5	22.750	26.043	3514
		Dogsleep	8	4.070	1.312	958
		Sleeping stage	59	2.070	1.049	3637
		Second Awakening	4	11.822	6.596	1329
	Schmitz OJ (2012)	Early Awakening	6	25.338	24.270	4736
		Dogsleep	16	3.837	1.315	1616
		Sleeping stage	38	2.578	1.459	2699
		Second Awakening	5	28.258	6.118	4177
	MacDonald PE (2006)	Early Awakening	4	17.739	14.095	2203
		Dogsleep	26	3.723	1.151	2916
		Sleeping stage	92	2.326	1.138	6342
		Second Awakening	12	13.239	6.151	4836
	Powell K (2007)	Early Awakening	7	24.797	28.778	5397
		Dogsleep	19	4.265	1.215	2097
		Sleeping stage	111	1.869	1.302	5800
		Second Awakening	4	6.017	0.599	678
	Alerstam T (2007)	Early Awakening	4	13.426	7.814	1673
		Dogsleep	20	3.578	1.006	1926
		Sleeping stage	67	2.480	0.950	4287
		Second Awakening	4	6.454	0.588	728
	Pan J (2007)	Early Awakening	5	19.542	17.815	3038
		Dogsleep	38	3.948	0.872	4554
		Sleeping stage	77	2.581	1.556	5889
		Second Awakening	4	6.768	0.246	813

As shown in the results of Spearman correlation analysis (Table 13), we found that there was a significant strong correlation between the Ab mean, standard deviation and Viewed of Dogsleep and the Ab standard deviation of the Sleeping stage, the Viewed of Dogsleep and the Ab standard deviation and Viewed of the Sleeping stage, the duration of Dogsleep and the mean of comprehensive influence of the Sleeping stage. What’s more, there was a significant moderate correlation between the Viewed of Dogsleep and its duration of Sleeping. These prove what the definition assumes about Dogsleep: the greater the Dogsleep intensity, the greater the Ab standard deviation in the Sleeping stage, and the more unstable the sleeping of A-SB. As for A-SB's awakening, we found that there was a significant moderate negative correlation between the Ab mean of Dogsleep and the Viewed of the Awakening stahe, the Ab standard deviation of the Sleeping stagge and the Ab mean of the Awakening stage, the viewed of Dogsleep and the Ab mean of the Awakening stage. This indicates that in the overall trajectory of A-SB, the distribution of Prince in Dogsleep and Dogsleep intensity have a negative effect on Awakening intensity and the scale of Prince, while the greater the Dogsleep intensity of A-SB, the less stable the sleeping. Therefore, we consider that the instability of A-SB’s sleeping will hinder the scale of Prince and the degree of attention after awakening to a certain extent. Additionally, the Viewed of Awakening presents a significant strong correlation with the duration and intensity of this stage, which once again demonstrates the leading role of Viewed in awakening A-SB.

**Table 13:** Spearman correlation coefficients (and their confidence intervals) between index information of each stage of A-SB and AA-SB.

Indicators		Dogsleep time	Sleeping time	Awakening time	Ab of Dogsleep	Ab of Sleeping	Ab of Awakening	Standard Deviation of Ab in Dogsleep	Standard Deviation of Ab in Sleeping	Standard Deviation of Ab in Awakening	Viewed of Dogsleep	Viewed of the Sleeping stage	Viewed value of the Awakening stage
Dogsleep time	Correlation Coefficient	1	0.625*	0.48	−0.146	0.774**	−0.396	−0.173	0.437	−0.178	0.825**	0.806**	0.014
	Sig. (double test)	.	0.04	0.135	0.669	0.005	0.228	0.611	0.179	0.601	0.002	0.003	0.968
Sleeping time	Correlation Coefficient	0.625*	1	0.31	0.292	0.192	−0.534	0.114	0.434	−0.251	0.731*	0.877**	−0.215
	Sig. (double test)	0.04	.	0.353	0.383	0.572	0.09	0.738	0.183	0.456	0.011	0	0.526
Awakening time	Correlation Coefficient	0.48	0.31	1	−0.493	0.249	0.475	−0.392	−0.198	0.544	0.157	0.3	0.760**
	Sig. (double test)	0.135	0.353	.	0.123	0.461	0.14	0.233	0.559	0.084	0.645	0.371	0.007
Ab of Dogsleep	Correlation Coefficient	−0.146	0.292	−0.493	1	−0.155	−0.518	0.782**	0.709*	−0.391	0.373	0.282	−0.618*
	Sig. (double test)	0.669	0.383	0.123	.	0.65	0.102	0.004	0.015	0.235	0.259	0.401	0.043
Ab of Sleeping	Correlation Coefficient	0.774**	0.192	0.249	−0.155	1	−0.345	0.036	0.518	−0.273	0.6	0.418	−0.091
	Sig. (double test)	0.005	0.572	0.461	0.65	.	0.298	0.915	0.102	0.417	0.051	0.201	0.79
Ab of Awakening	Correlation Coefficient	−0.396	−0.534	0.475	−0.518	−0.345	1	−0.482	−0.673*	0.873**	−0.682*	−0.6	0.0891**
	Sig. (double test)	0.228	0.09	0.14	0.102	0.298	.	0.133	0.023	0	0.021	0.051	0
Standard Deviation of Ab in Dogsleep	Correlation Coefficient	−0.173	0.114	−0.392	0.782**	0.036	−0.482	1	0.727*	−0.582	0.273	0.127	−0.582
	Sig. (double test)	0.611	0.738	0.233	0.004	0.915	0.133	.	0.011	0.06	0.417	0.709	0.06
Standard Deviation of Ab in Sleeping	Correlation Coefficient	0.437	0.434	−0.198	0.709*	0.518	−0.673*	0.727*	1	−0.6	0.791**	0.591	−0.555
	Sig. (double test)	0.179	0.183	0.559	0.015	0.102	0.023	0.011	.	0.051	0.004	0.056	0.077
Standard Deviation of Ab in Awakening	Correlation Coefficient	−0.178	−0.251	0.544	−0.391	−0.273	0.873**	−0.582	−0.6	1	−0.5	−0.382	0.873**
	Sig. (double test)	0.601	0.456	0.084	0.235	0.417	0	0.06	0.051	.	0.117	0.247	0
Viewed of Dogsleep	Correlation Coefficient	0.825**	0.731*	0.157	0.373	0.6	−0.682*	0.273	0.791**	−0.5	1	0.927**	−0.382
	Sig. (double test)	0.002	0.011	0.645	0.259	0.051	0.021	0.417	0.004	0.117	.	0	0.247
Viewed of the Sleeping stage	Correlation Coefficient	0.806**	0.877**	0.3	0.282	0.418	−0.6	0.127	0.591	−0.382	0.927**	1	−0.273
	Sig. (double test)	0.003	0	0.371	0.401	0.201	0.051	0.709	0.056	0.247	0	.	0.417
Viewed value of the Awakening stage	Correlation Coefficient	0.014	−0.215	0.760**	−0.618*	−0.091	0.891**	−0.582	−0.555	0.873**	−0.382	−0.273	1
	Sig. (double test)	0.968	0.526	0.007	0.043	0.79	0	0.06	0.077	0	0.247	0.417	.

The correlation is significant at a confidence level (double test) of 0.05.

The correlation is significant at a confidence level (double test) of 0.01.

All the indexes are broken down by “Dogsleep time,” “Sleeping time−Awakening time,” “Ab of Dogsleep,” “Ab of Sleeping,” “Ab of Awakening,” “Standard Deviation of Ab in Dogsleep,” “Standard Deviation of Ab in Sleeping,” “Standard Deviation of Ab in Awakening,” “Viewed of Dogsleep,” “Viewed of the Sleeping stage,” and “Viewed value of the Awakening stage.”

#### Influence of A-SB’s bibliometric attributes on awakening

Previous researches have studied in detail the relationship between the bibliometric attributes and diffusion trajectories of SBs. In order to explore whether the bibliometric attributes of A-SB are related to their awakening and whether they have some critical features of C-SB, we try to mine some important bibliometric attributes of A-SB (Table 14).

**Table 14:** Bibliometric attributes of A-SB in *PLOS Biology*.

	Text attributes						
	Document	Document type	Title length	Text length	References	Number of authors	Number of keywords
A-SB	Neugebauer KM(2006)	Editorial Material	9	1918	7	1	1
	Tsuriel S(2006)	Article	11	14664	69	8	10
	Del Cul A(2007)	Article	10	13969	66	3	10
	DeRisi S(2003)	Editorial Material	6	1653	1	3	1
Aa-SB	Montooth KL(2008)	Editorial Material	8	3908	29	2	9
	Khalturin K(2008)	Article	10	10900	47	6	10
	Schmitz OJ(2012)	Editorial Material	4	2297	14	2	2
	MacDonald PE(2006)	Editorial Material	10	4124	43	2	10
	Powell K(2007)	Editorial Material	4	5347	11	1	4
	Alerstam T(2007)	Article	9	6371	34	5	10
	Pan J(2007)	Editorial Material	8	3565	19	2	10

Through the correlation analysis of A-SB’s bibliometric attributes and the indicators in its Awakening stage (Table 15), we discovered that there was no correlation between the Prince of 11 A-SB—Viewed, the Ab of the Awakening stage and the bibliometric attributes, that there was a significant medium positive correlation between Discussed and the number of authors, and that Citations presented a strong positive correlation with A-SB’s title length, text length, number of references, and number of keywords. This reflects that, on the one hand, the longer the title and text of A-SB, the more documents cited; the more extensive the research topics involved, the more citations can be obtained in the Awakening stage, which is similar to C-SB, although Citations does not make a difference during the awakening process of 11 A-SB. On the other hand, it shows that the diffusion trajectory of scientific literature reflected by Ab is not consistent with the citation trajectory. Ab can measure the influence of literature from two aspects of citation diffusion and social media diffusion, and identify A-SB. Meanwhile, the greater number of authors is conductive to A-SB getting more communication and discussion in Awakening.

**Table 15:** Correlation analysis of various indicators in the Awakening stage and text attributes.

Indicators		Title length	Text length	References	Number of authors	Number of keywords
Citations	Correlation Coefficient	0.710*	0.510	0.830**	0.903**	0.663*
	Sig.	0.014	0.109	0.002	0.000	0.026
Saved	Correlation Coefficient	−0.116	−0.103	0.229	0.219	0.057
	Sig.	0.734	0.763	0.499	0.517	0.867
Discussed	Correlation Coefficient	0.430	0.623*	0.240	0.362	0.521
	Sig.	0.187	0.041	0.478	0.274	0.100
Viewed	Correlation Coefficient	−0.365	−0.266	−0.027	0.000	0.149
	Sig.	0.270	0.428	0.937	1.000	0.662
Ab of Awakening	Correlation Coefficient	−0.342	−0.280	−0.009	0.027	0.149
	Sig.	0.304	0.403	0.979	0.937	0.662

The correlation is significant at a confidence level (double test) of 0.05.

The correlation is significant at a confidence level (double test) of 0.01.

Viewed numerically, although Viewed and Ab have negative correlation coefficients with title length, number of authors and text length, they are not significantly correlated. It indicates that users, who comprise the public as main body, generally pay little attention to the length of title and text of the documents when browsing academic papers, which is inconsistent with the tendency of academic researchers when choosing documents.

## Conclusions

This study extends SBs from a research perspective based on citation indicator diffusion trajectory to a comprehensive evolution trajectory research based on Altmetrics indexes. The research identified its Prince based on the recognition of A-SB, explored the specific reasons for the Awakening of A-SB, and excavated awakening mechanism of A-SB from the three dimensions of the influence between the indicators, the overall evolution trajectory of A-SB, and literature bibliometric attributes, making the SBs and identification of its Prince more accord with the actual diffusion and evolution of the scientific literature. During the research process, the following conclusions were mainly drawn:

1. The study, which defines SBs princes under the Altmetrics perspective, points out the new characteristics of the Prince on social media platforms. Under the social media platform, the awakening of A-SB is realized under the action of a single indicator or the combination of multiple indicators. However, there is bound to be a decisive indicator with the largest proportion, which makes A-SB receive high attention after a long Sleeping period and reflects the most prominent academic or social behavior that awakens and sustains the Awakening of A-SB. Based on these features, we described the Awakening trajectory of SBs through quantitative Ab index, and recognized A-SB and its Prince from a new perspective.
2. Based on Ab index, this study identifies four A-SB literature and seven Aa-SB literature among 3541 literature in the journal *PLOS Biology*, and conducts empirical research on Prince identification based on this literature. We found that there are two awakening methods in the 11 literature during Delayed Recognition stage, and users’ browsing and downloading of the information of online literature is considered as Prince, which can often awaken A-SB alone and make the greatest contribution in the way of awakening in cooperation with other indicators.
  Different from the awakening mechanism of SBs under the view of traditional citation, Citation does not dominate the awakening of A-SB but, together with Saved, plays the role of assisting Prince to maintain the Awakening of A-SB. In addition, A-SB is rarely discussed or evaluated by researchers on social networking sites during its Awaking stage, which is closely related to the number of co-authors of A-SB. Nor has it been officially endorsed by researchers. These, on the one hand, reflect the importance of the dimension of social influence in A-SB’s awakening mechanism. Academic researchers are not the main body to awaken A-SB. On the other hand, these fully reflect that adding Altmetrics indexes into the study of SBs is a beneficial supplement, making the awakening mechanism of SBs more comprehensive and scientific.
3. This study reveals the internal mechanism within A-SB awakening mechanism from three different dimensions, and finds that during the awakening process of A-SB, there is a dynamic relationship between the indicators in the Awakening stage and the stages under the overall trajectory. We discovered that the spread between two different users’ social behaviors—users’ browsing and downloading of online literature information and users’ behavior of saving information in the literature manager such as adding bookmarks—has a significant effect on the Awakening of A-SB, an interesting phenomenon similar to that of Prince and his squire in the awakening mechanism of SBs under traditional citation trajectory. In the trajectory of Delayed Recognition stage of A-SB, we define the Dogsleep of SBs, which mirrors that the instability of the Sleeping of SBs will generate a certain negative impact on Prince of A-SB and Awakening intensity. It is also a supplement to the diffusion trajectory of the SBs. Besides, the title, length, the number of references and the amount of the topics involved are all conducive to the accumulation of Citations in the Awakening process of A-SB, however, from the point of Altmetrics, they cannot reflect the tendency of users to read academic papers, which again proves that traditional citation index cannot be neglected in the awakening mechanism of A-SB. These findings indicate that the awakening mechanism of A-SB is both related to and new to SBs in the traditional citation trajectory.

## Discussions

In the traditional citation evolution trajectory, the Citations represents the academic influence of scientific literature. Contrarily, on social media platforms, Altmetrics indexes can not only reflect the social influence of scientific literature but also effectively evade the highly cited phenomenon caused by fake citations and the Matthew effect [87], thereby improving the accuracy of identifying literature with trajectory under the comprehensive evolution trajectory. However, some researchers believe that Altmetrics primarily reflects “attention” rather than influence or influence force [37,67]. When combing the dynamic distribution of the indicators of A-SB, we also observed that early surge existed in the Altmetrics indexes because, as an OA platform, PLOS access to literature is not subject to subscription, and researchers tend to view the latest published papers online, which can accumulate Viewed quickly in the early days of publication. Maybe this phenomenon should be explained from the perspective of “attention.” Therefore, from the point of Viewed, the long-tailed phenomenon of the time distribution of Viewed of A-SB denotes a rapid decline in user’s attention to them, that is, A-SB will enter into Sleeping stage because of being quickly forgotten. During Sleeping, the Ab of A-SB will reach a state that is neither fully sleeping nor truly awakening, causing it to fall into Dogsleep (similar to “heartbeat” [29]. Owing to the short duration, it occupies less proportion in the evolution trajectory of SBs, but it does not mean that its existence should be ignored, especially the prediction research of SBs. Besides, regarding the awakening of A-SB, we found the significance of Viewed as Prince. However, browsing behaviors on social media platforms include both web browsing and literature resource downloading, and the latter is obviously a more valuable use of literature. Thus, the problem that whether the effectiveness of Viewed on the Open Access Platform as a scoring indicator of the comprehensive evolution trajectory of scientific literature be recognized still remains. Besides, the interesting question is that A-SB is a supplement to the scientific Sleeping Beauty research, which, like the traditional sleeping beauty, would receive continuous attention after awakening. However, will A-SB receive continuous and more attention or reference after the Delay Recognition stage? This is related to the second stage of the diffusion track of literature, which is worth exploring. Moreover, despite interesting phenomena between the bibliometric attributes of the literature and the trajectory of the Awakening stage, some cannot be considered as effective conclusions due to the sample quantity of A-SB. The questions above need to be further discussed and studies.

This study has some limitations worth acknowledging. First, during the collection and evaluation of the citations index, considering that we currently cannot obtain monthly citation information for each literature in a longer interval in the Web of Science database, we adopted the average annually cited data of each literature on a monthly basis, which could have a certain impact on our research results. However, this impact will not cause major changes to the study results. In the future, with the gradual improvement of the development of Open Access, this issue will be solved. To date, various scholarly data have easily accessed and powerful data analysis technologies being developed, which will also help us to develop and improve new perspectives of scientific research [88]. Second, the disciplines covered by the sample journals are relatively single, and the data are generated from the published literature of *PLOS Biology* from 2003 to 2019. The disciplines are concentrated in the field of biology. In this case, whether the analysis of bibliometric attribute dimensions in the research has universal applicability remains unclear. However, we believe this issue could affect the conclusion of bibliometric attributes but not the trajectory of A-SB (the latter is our main concern), because our parameters are set according to the overall situation of the sample. Thus, with the expansion of the total number of samples, the parameters will be improved; this would help to improve our current conclusion, but it does not imply that our conclusion is completely invalid. Finally, in this study, the scope of our discussion is only the trajectory in Delayed Recognition stage of SBs, but we found during the research that some literatures have multi-stage Awakening with >3 stages. The question arises, does the Prince of that literature with multi-stage Awakening have a new feature? There is no denying that the breadth of the A-SB research still requires further expansion.

In the future, with the promotion and improvement of Open Access Platform, the information of journal literatures will be more abundant, and extending the time window of the literature will become feasible. Thus, we will focus on exploring the validity of the characteristic of the Prince of SBs under the perspective of Altmetrics in an interdisciplinary context after the expansion of data samples, and further improve the awakening mechanism of SBs.

## Supporting information

S1 Fig Neugebauer (2006). (TIF)

S2 Fig Tsuriel (2006). (TIF)

S3 Fig Del Cul (2007). (TIF)

S4 Fig DeRisi (2003). (TIF)

S5 Fig Montooth (2008). (TIF)

S6 Fig Khalturin (2008). (TIF)

S7 Fig Schmitz (2012). (TIF)

S8 Fig MacDonald (2006). (TIF)

S9 Fig Powell (2007). (TIF)

S10 Fig Alerstam (2007). (TIF)

S11 Fig Pan (2007). (TIF)
