# Supplementary figures and images for: Identifying the princes base on Altmetrics: An awakening mechanism of sleeping beauties from the perspective of social media
Source: PLoS One. 2020 Nov 25;15(11):e0241772. doi: 10.1371/journal.pone.0241772 (PMC7688316; doi:10.1371/journal.pone.0241772)

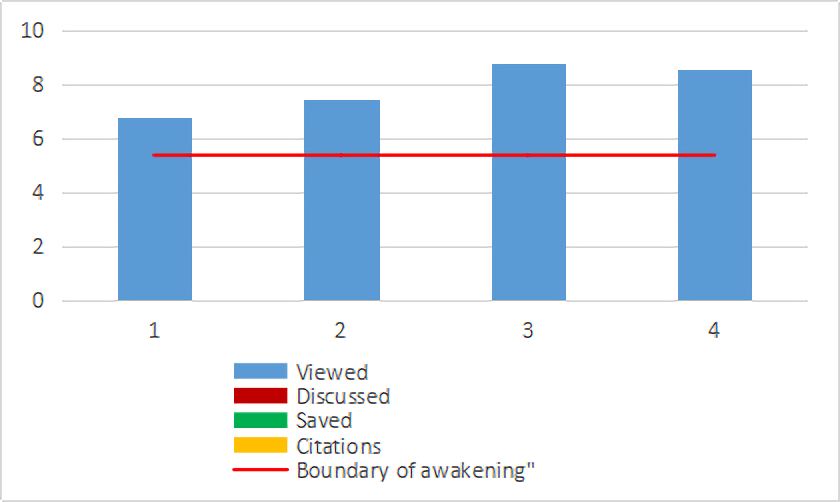

Supplement: S1 Fig — (TIF) [file pone.0241772.s001.tif]

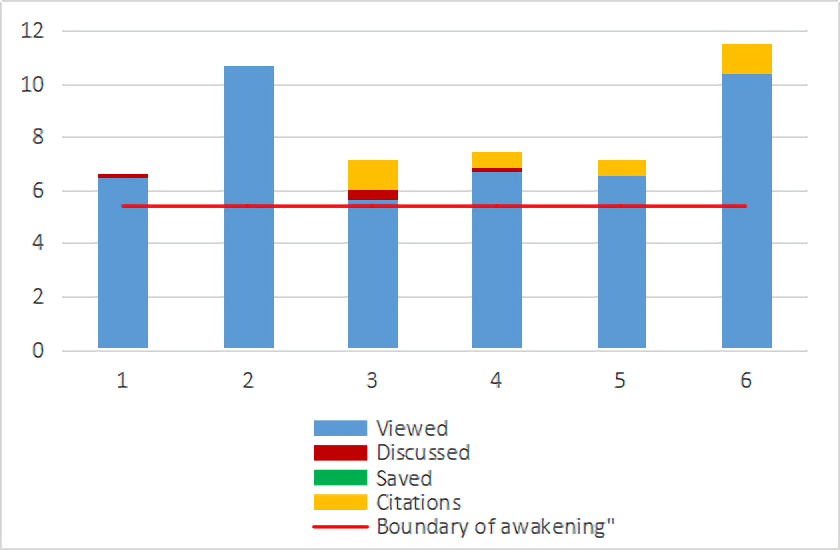

Supplement: S2 Fig — (TIF) [file pone.0241772.s002.tif]

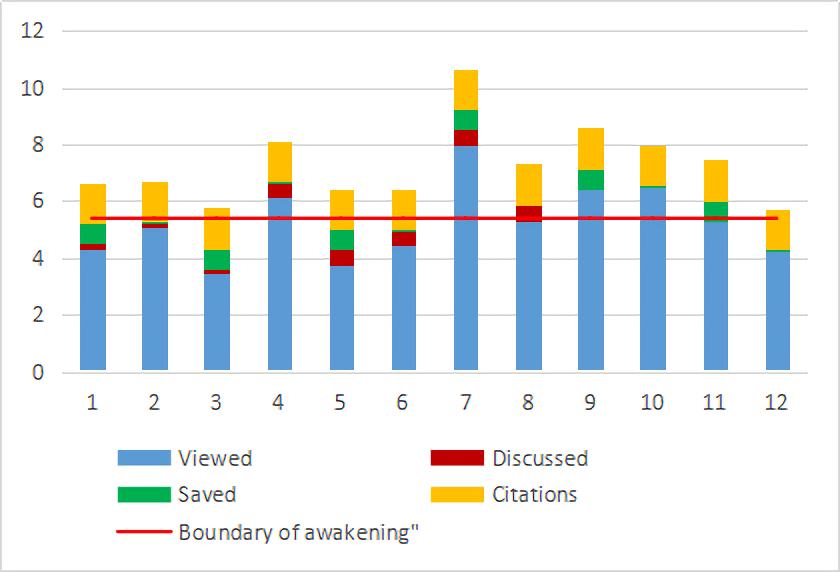

Supplement: S3 Fig — (TIF) [file pone.0241772.s003.tif]

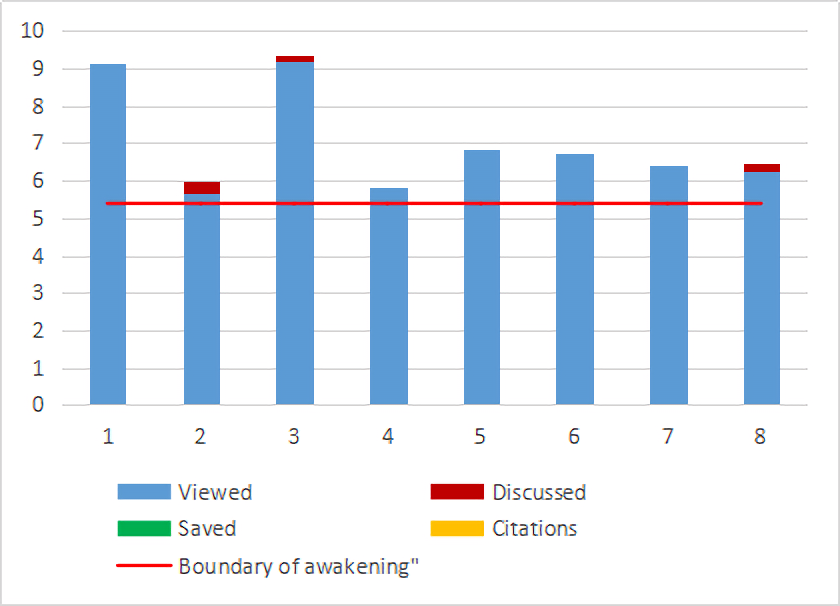

Supplement: S4 Fig — (TIF) [file pone.0241772.s004.tif]

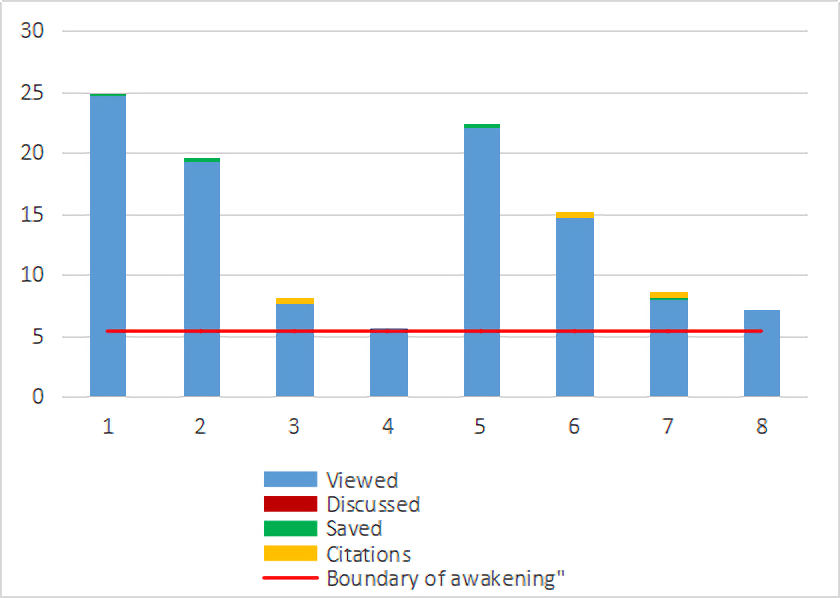

Supplement: S5 Fig — (TIF) [file pone.0241772.s005.tif]

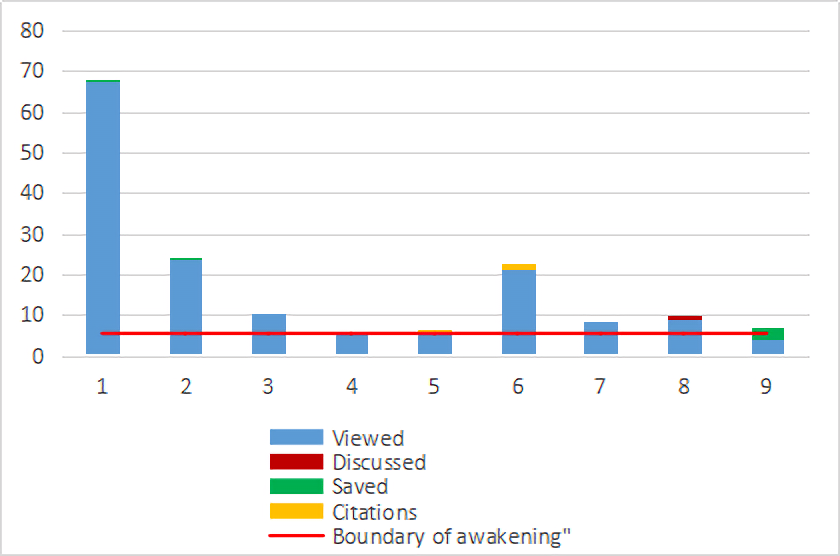

Supplement: S6 Fig — (TIF) [file pone.0241772.s006.tif]

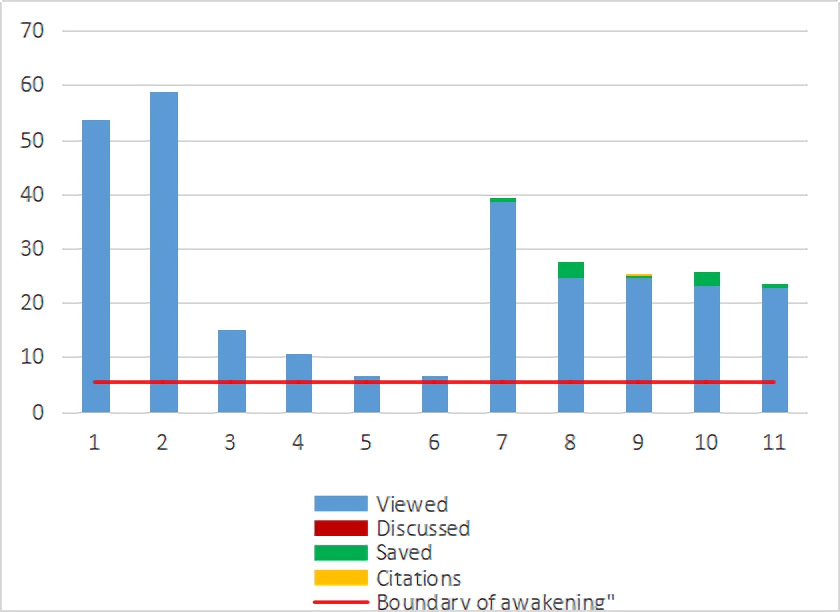

Supplement: S7 Fig — (TIF) [file pone.0241772.s007.tif]

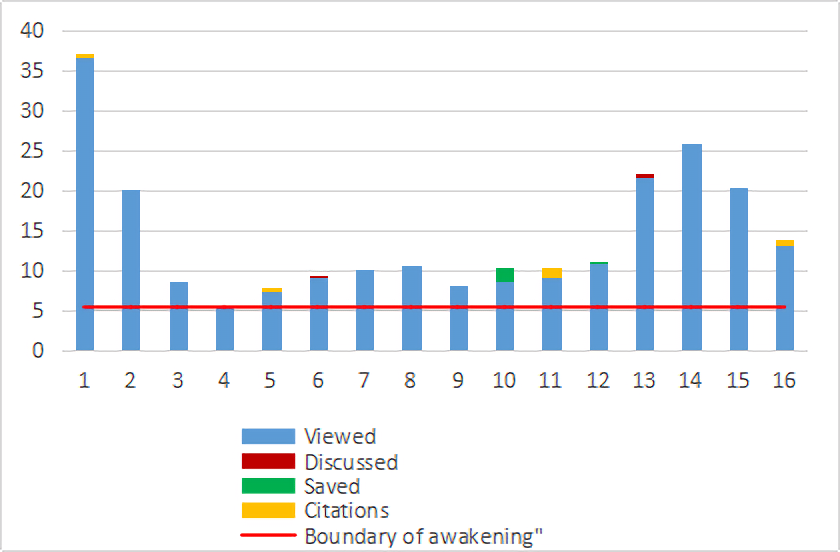

Supplement: S8 Fig — (TIF) [file pone.0241772.s008.tif]

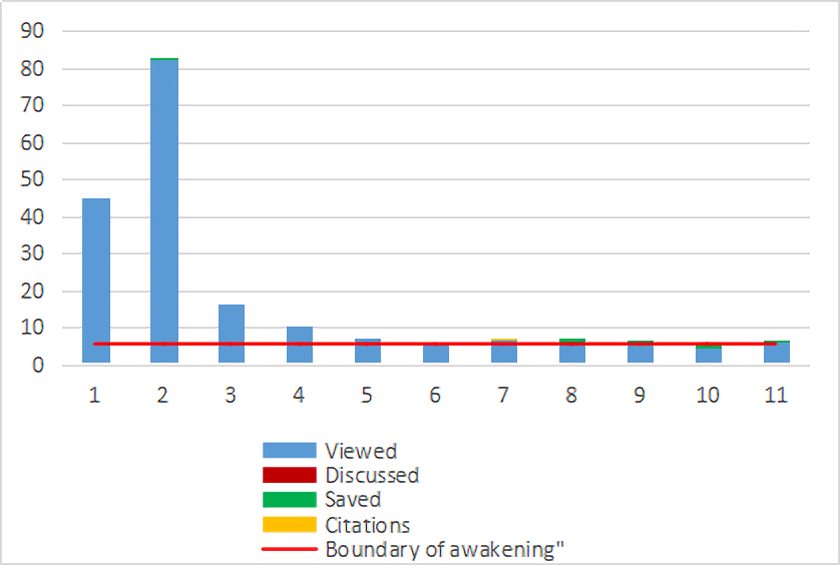

Supplement: S9 Fig — (TIF) [file pone.0241772.s009.tif]

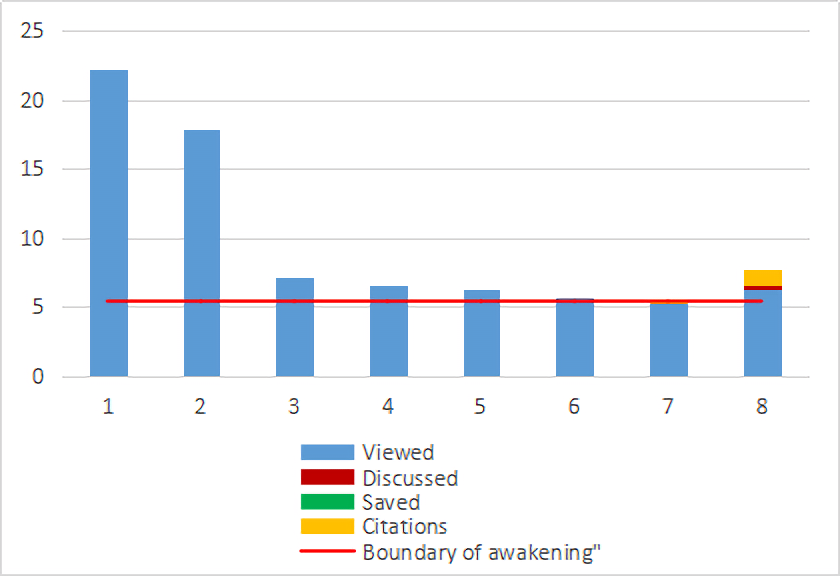

Supplement: S10 Fig — (TIF) [file pone.0241772.s010.tif]

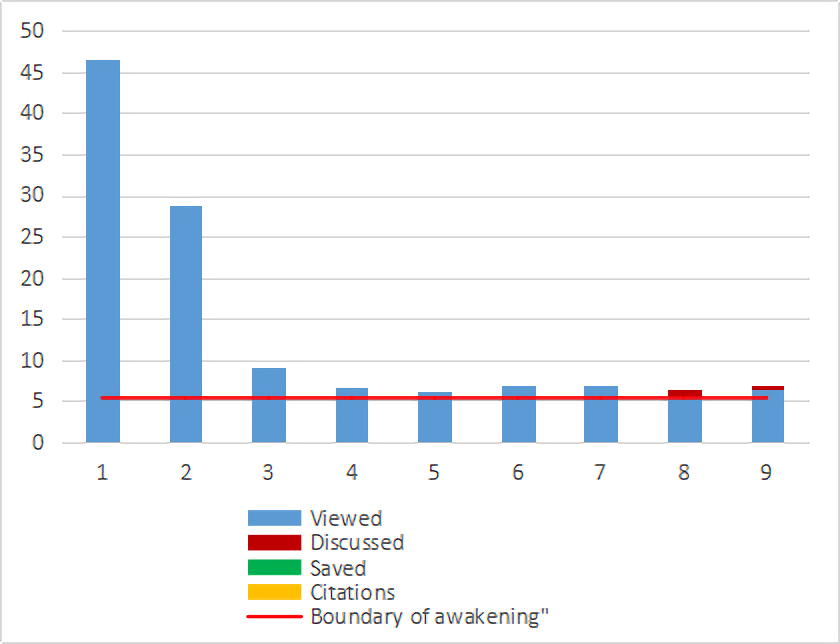

Supplement: S11 Fig — (TIF) [file pone.0241772.s011.tif]
